# Diverse sources of reward value signals in the basal ganglia nuclei transmitted to the lateral habenula in the monkey

**DOI:** 10.3389/fnhum.2013.00778

**Published:** 2013-11-13

**Authors:** Simon Hong, Okihide Hikosaka

**Affiliations:** ^1^Department of Brain and Cognitive Sciences, McGovern Institute for Brain Research, Massachusetts Institute of TechnologyCambridge, MA, USA; ^2^Laboratory of Sensorimotor Research, National Eye Institute, National Institutes of HealthBethesda, MD, USA

**Keywords:** orthodromic stimulation, patchy activation, striosome, patch, matrix, striatum, dopamine, motivation

## Abstract

The lateral habenula (LHb) plays an important role in motivational decision making. Neurons in the primate LHb signal negative ‘reward prediction errors’ and inhibit midbrain dopamine (DA) neurons. These negative reward prediction error signals in the LHb are, at least partly, provided by a distinct group of neurons in the border region of the globus pallidus internal segment (GPb). However, it is still unclear whether other basal ganglia nuclei provide the LHb with reward signals, either through the GPb or through different circuits. As a first step to answer this question, we electrically stimulated various parts of the basal ganglia and monitored the neural activity in the LHb in the awake monkey. First, we found that low intensity stimulations in the GPb and the internal segment of the globus pallidus (GPi) evoked a short latency (5 ms) excitatory response in LHb neurons. Second, LHb neurons were inhibited by stimulations in the ventral pallidum (VP). These results suggest that reward-related signals are transmitted to the LHb mainly through excitatory connections from the GPb and inhibitory connections from the VP. Finally, excitations or inhibitions are induced in LHb neurons from diverse but patchy regions in the striatum. These effects have considerably longer latencies, suggesting that they may be mediated by the GPb or the VP. The patchy nature of the stimulation effect raises the possibility that the striosomes are the source of reward-related signals transmitted to the LHb.

## Introduction

The lateral habenula (LHb) is involved in fundamental aspects of animal behavior (Hikosaka, 2010). In particular, its role in motivational and emotional processes, as well as their disorders, has been a focus of recent research (e.g., Matsumoto and Hikosaka, 2007; Brown et al., 2010; Sartorius et al., 2010; James et al., 2011; Li et al., 2011; Shabel et al., 2012; Stamatakis and Stuber, 2012). Neurons in the primate lateral LHb are excited by visual stimuli that predict the absence of reward and are inhibited by stimuli that predict the presence of reward (Matsumoto and Hikosaka, 2007), and inhibit dopamine (DA) neurons in the midbrain (Lisoprawski et al., 1980; Christoph et al., 1986; Ji and Shepard, 2007; Matsumoto and Hikosaka, 2007), mainly through the rostromedial tegmental nucleus (RMTg; Jhou et al., 2009; Hong et al., 2011; Barrot et al., 2012).

In our previous study (Hong and Hikosaka, 2008a) we showed that, (1) neurons in the border region of the globus pallidus internal segment (GPb) projected their axons to the LHb, (2) most of the LHb-projecting GPb neurons encoded negative reward prediction errors similar to LHb neurons, and (3) their reward-related activity started earlier than the activity of LHb neurons. These results suggest that the GPb-LHb connection is excitatory, although a minority of GPb neurons encoding positive reward prediction errors may have inhibitory connections to the LHb (Hong and Hikosaka, 2008a). This hypothesis was supported by studies using rats (Shabel et al., 2012) and lampreys (Stephenson-Jones et al., 2013).

How then does the GPb-LHb circuit acquire the signals that are necessary to create reward prediction error signals? One candidate for the source of such signals is the striatum. Physiologically, it is known that many striatal neurons show sensory responses that are modulated by expected reward values (Kawagoe et al., 1998; Oyama et al., 2010; Ambroggi et al., 2011), or exhibit sustained activity predicting sensory stimuli, actions, or reward itself (Hikosaka et al., 1989; Tremblay et al., 1998; Lauwereyns et al., 2002; Lau and Glimcher, 2008; Hori et al., 2009). Anatomically, some studies have suggested that the striatum sends signals to the LHb through indirect connections. In rats, Rajakumar et al. (1993) reported that the rostral part of the entopeduncular nucleus (EPN), which showed strong connections to the LHb, received projections from the striatum, particularly from the striosome (Graybiel and Ragsdale, 1978). In monkeys, using a manganese tracer method, Saleem et al. (2002) found that the striatum had indirect but strong projections to the LHb. However, it was unknown whether the striataum-LHb connection was mediated by the GPb. There may be other routes for the striatum to influence the LHb.

To examine the hypothesis that the striatum is the origin of the input to the LHb, we electrically stimulated various parts of the basal ganglia while recording from the LHb. The spatial distribution of the effective stimulation sites as well as the latency and the direction of modulation of the LHb activity provide suggestions for the functional circuits connecting the basal ganglia to the LHb. An initial result of this study was presented in the SfN abstract (Hong and Hikosaka, 2008b).

## Materials and Methods

Three rhesus monkeys (Macaca mulatta), B, C and D, were used as subjects in this study. All animal care and experimental procedures were approved by the National Eye Institute and Institutional Animal Care and Use Committee and complied with the Public Health Service Policy on the humane care and use of laboratory animals.

### Electrophysiology

One recording chamber was placed over the midline of the occipital cortex, tilted posteriorly by 40°, and was aimed at the LHb; another chamber for the stimulating electrode was placed over the frontoparietal cortex, tilted laterally by 35°, and was aimed at the GPi. Recordings and electrical stimulations were performed using tungsten electrodes (Frederick Haer) that were advanced by an oil-driven micromanipulator (MO-97A, Narishige). The recording and stimulation sites were determined using a grid system, which allowed recordings at every 1 mm between penetrations. An electrode was introduced into the brain through a stainless steel guide tube, which was inserted into one of the grid holes and then to the brain via the dura. We preferentially recorded multi-unit activity in the LHb using electrodes with low impedances (about 0.5 M Ohm). The signal was amplified with a band-pass filter (200 Hz–5 kHz; BAK, Mount Airy, MD) and collected at 40 kHz via a custom-made window discriminator (MEX). Single neurons were isolated online using a custom voltage-time window discrimination software (MEX, LSR/NEI/NIH).

**Figure 1:**
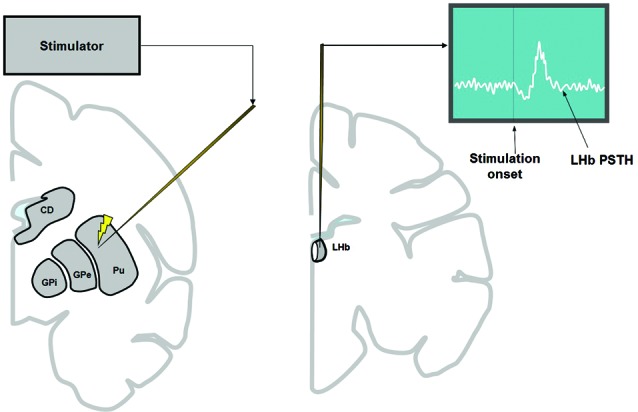
**To examine the influence of the basal ganglia on the LHb, electrical stimulation was applied in the basal ganglia (left) while recording multiunit activity from the LHb (right).** Stimulation was applied every 200 μm along the track of the stimulating electrode (left), 100 times at each depth. The stimulation was either 1 biphasic pulse of 100 μA (for all structures of basal ganglia) or 3 biphasic pulses of 133 μA each (only for striatum).

To record LHb activity, the position of the LHb was mapped first by MRI. The electrophysiological features of the LHb (Matsumoto and Hikosaka, 2007) were also used to locate the LHb. To examine the basal ganglia’s influence on the LHb, electric stimulations were applied to the basal ganglia while recording multi-unit activity from the LHb (Figure 1). In most cases we used 1 biphasic pulse of 100 μA lasting 0.2 ms. We chose these parameters because it was shown previously that stimulation with similar parameters in the CN in awake monkeys produced significant effects on single neurons in the substantia nigra pars reticulata (Hikosaka et al., 1993). However, single pulse stimulation in the striatum often elicited only weak effects on LHb neurons. For this reason, we used three biphasic pulses of 133 μA each lasting 0.2 ms at 300 Hz (for the striatum only). The inter-stimulation interval was about 1 sec with some variability. The stimulation-triggered LHb activity was stored and analyzed offline. To see the effect of stimulation at different structures of the basal ganglia, stimulations were applied 100 times at every 200 μm along the electrode penetration. To minimize stimulation-induced electrical artifacts, we used a commercially available artifact remover (Artifact Zapper-1, Riverband Instrument).

### Data analysis

**Figure 2:**
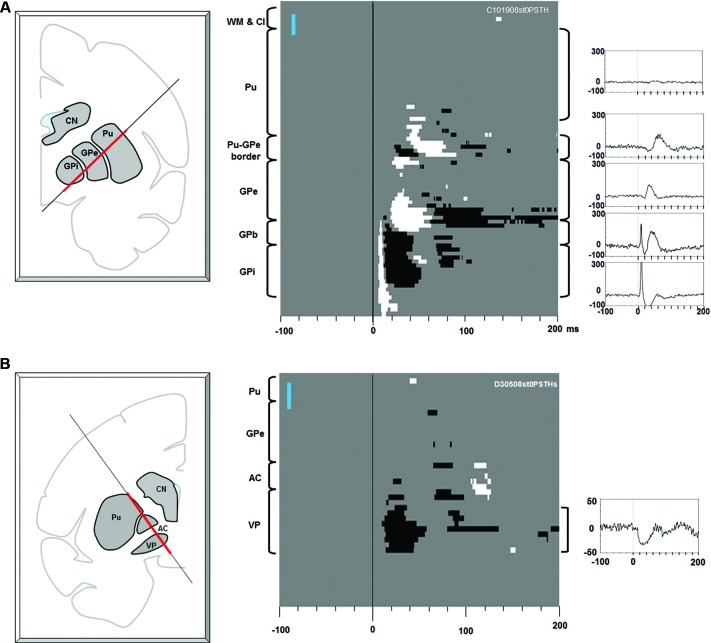
**Effects of electrical stimulation along electrode tracks through the putamen-pallidal region.** **(A)** Stimulations along the putamen-globus pallidus external segment (GPe)-GPi track. Left: The location of the stimulating electrode track. The red line indicates where the stimulation data were collected. Center: Significant effects (white: excitation, black: inhibition) shown in depth (ordinate) and time (abscissa). At each depth of stimulation, the LHb neuron activity in a pre-stimulation period (duration: 200 ms) was analyzed to generate a 95% confidence threshold. This threshold was used to determine a significant post-stimulation deviation of the LHb activity. The anatomical landmarks are indicated on the left side of the figure (WM: white matter; Cl: claustrum). The vertical blue bar indicates 1 mm. Right: Average LHb activity (baseline subtracted PSTH) in the structure corresponding to the bracket next to each peri-stimulus time histogram (PSTH). **(B)** Stimulations along the putamen-VP track. The same convention as in **(A)**.

**Figure 3:**
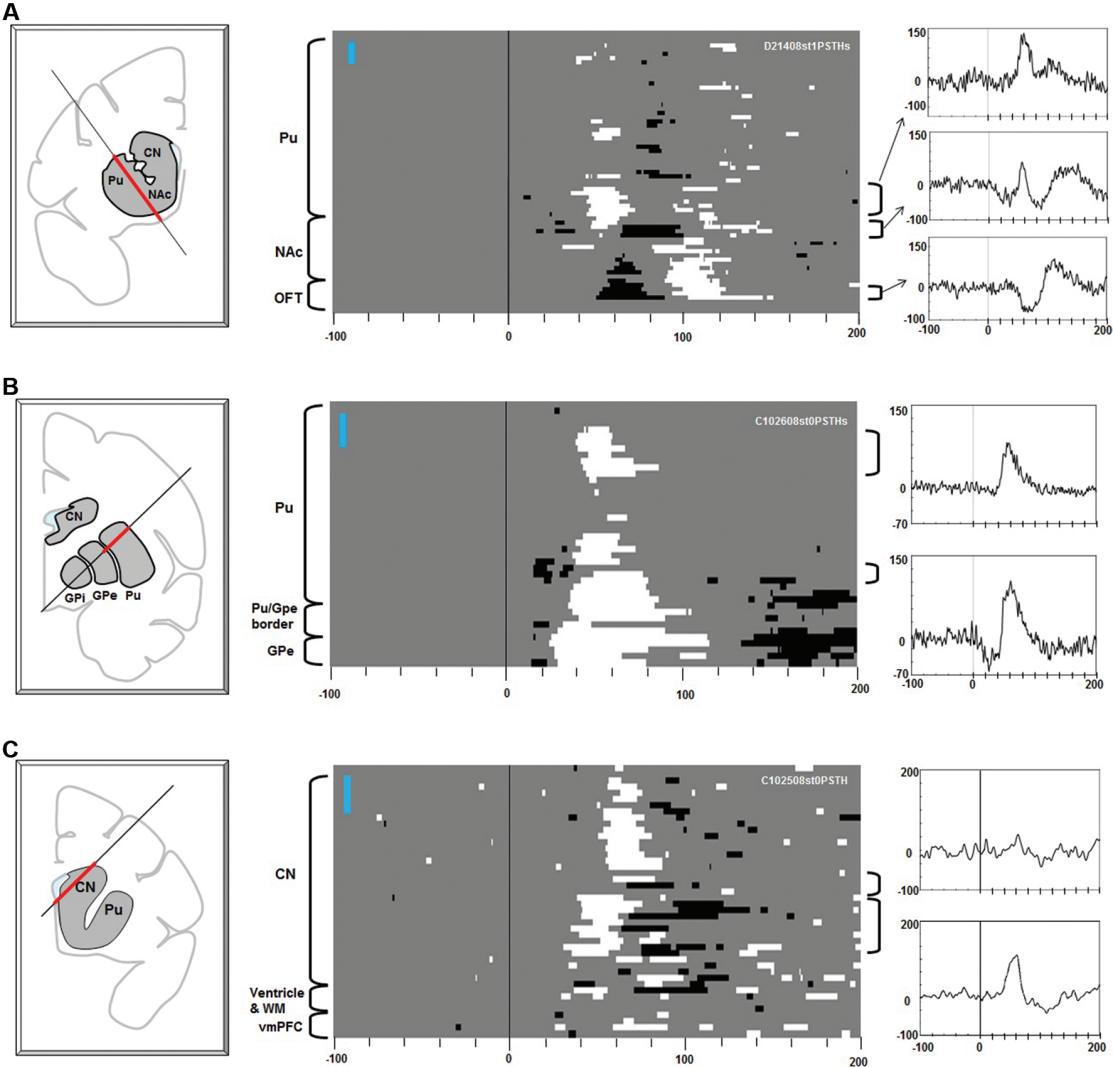
**Effects of stimulation along the electrode tracks through the striatum.** Electric stimulations of 1 biphasic pulse of 100 μA were used in **(A)**. Three pulse stimulations (3 biphasic pulses, 133 μA each) were used for **(B)** and **(C)**. The same convention as in Figure 2.

At the end of each penetration, a map of effective stimulation sites was made by averaging the 100 times of stimulating effect at every 200 μm along the whole tract. At each depth of stimulation, LHb neuron activity during the 200 ms period preceding the stimulation onset was analyzed to generate a 95% confidence threshold. The threshold was used to determine the significant deviation of LHb activity after stimulation. In the center figures of Figures 2 and 3, significant excitations are shown in white and significant inhibitions are shown in black.

To determine the latency of the stimulation-induced response of LHb neurons, we used a Poisson distribution test (Hong et al., 2011). For this, we first counted the number of accumulated spikes across the trials within a 1 ms bin along the 500 ms period before stimulation. Using these data, a histogram was constructed, with the abscissa representing the number of spikes and the ordinate representing the number of bins that had the number of spikes corresponding to the values on the abscissa. The histogram was fitted with a Poisson distribution curve. Using the Poisson curve, the threshold value of spikes per bin was determined that matched the *p* value of 0.01. Then, the number of spikes of each bin during the post-stimulation period was examined. The point in time when the histogram exceeded the significance level was taken to be the time of significant modulation by the stimulation.

## Results

To examine the basal ganglia’s influence on the LHb, electric stimulation was applied in the basal ganglia while recording multi-unit activity from the LHb. Across the experiments, the stimulating electrode was aimed at different positions of the striatum, while the recording electrode was aimed at the LHb on the same side. We monitored multi-unit activity, rather than single-unit activity, for two reasons. First, LHb neurons show very similar activity in relation to changes in reward values (Matsumoto and Hikosaka, 2007). Second, multi-unit activity remained more stable even when the recording lasted several hours.

Figure 2A shows the effects of electrical stimulations (one bi-phasic 100 μA pulse of 0.2 ms duration) along the electrode track traveling through the putamen, GPe, and GPi. The stimulation effect changed dramatically with the depth of the stimulating electrode. Stimulations in the putamen induced little effect. As the stimulating position moved closer to the border part between the putamen and GPe, a small excitatory effect appeared about 35 ms after the stimulation. As the stimulating electrode approached the GPe, inhibitory and then excitatory effects appeared with shorter latencies (about 25 ms). In the dorsal part of the GPe, however, there was little stimulation effect. In the ventral GPe, an excitatory effect appeared at about 20 ms. Interestingly, as soon as the electrode came out of the GPe and entered the GPe-GPi border region (GPb), a strong phasic excitatory response occurred with a short latency (5 ms), which was followed by an inhibition. The phasic excitation continued throughout the GPi and further into a region ventral to the GPi (which may also belong to the GPb). The data suggest that LHb neurons receive excitatory or inhibitory inputs from different regions in the basal ganglia and that their sources were distributed in a patchy manner.

Another source of short latency responses was found in the VP (Figure 2B). The stimulating electrode passed along the lateral edge of the putamen, the anterior part of the GPe, the anterior commissure (AC), and reached the VP. The stimulation effect was mostly confined in the VP and was an inhibitory response that started about 15 ms after the stimulation and lasted about 40 ms.

Stimulation in the striatum elicited diverse effects in the multi-unit LHb activity (Figure 3). Excitatory responses were evoked from patchy areas in the putamen, in its rostral portion (Figure 3A) and central portion (Figure 3B) (latencies: 30–40 ms). Inhibitory responses with shorter latencies (15–25 ms) were evoked from the ventral portion of the central putamen, close to the transition to the GPe (Figures 2A, 3B). Stimulations in the ventral striatum, including the nucleus accumbens (NAc), evoked inhibitory responses (latencies: 50–70 ms) followed by an excitation (Figure 3A, center). Stimulations in the rostral portion of the caudate nucleus (CN) evoked excitatory responses with different latencies (dorsal CN: 60 ms, ventral CN: 30–40 ms). Overall, the effective sites of stimulation were distributed in a patchy manner in the striatum. Their effects (i.e., combination of excitatory and inhibitory effects) and latencies were variable across the patches.

## Discussion

### GPb-LHb connection

The data in Figure 2A suggest that LHb neurons receive excitatory and inhibitory inputs from different regions in the basal ganglia. The short latency response evoked from the GPb-GPi region may be explained by the connection from the GPb to the LHb (Parent et al., 1981). We previously found that many GPb neurons projected their axons to the LHb and a majority of them showed negative reward modulations of their responses to visual saccade targets (Hong and Hikosaka, 2008a). Since LHb neurons also showed similar negative reward modulations, the GPb-LHb connection was considered to be excitatory. Our present data (Figure 2A) are consistent with this hypothesis. The excitatory nature of the GPb-LHb connection was supported by recent studies in rats (Barroso-Chinea et al., 2008; Shabel et al., 2012) and lampreys (Stephenson-Jones et al., 2013).

The results in Figure 2A reflect the functional anatomy reported in the literature. The short latency excitatory effect was observed not only from the GPb but also from the underlying areas including the GPi and an area below it. This may be due to the anatomical structure of the GPb. Neurons that project to the LHb are located not only in the GPb but also in the middle of the GPi (close to the accessory medullary lamina, AML) and, occasionally, below the border area of the GPi (Hong and Hikosaka, 2008a). If the GPb is defined as the area that connects to the LHb, it has a complex 3D layered structure (see also Parent et al., 2001).We also speculate that the axons of the GPb neurons travel across these layers (i.e., top layer: GPe-GPi border, middle layer: AML, bottom layer: ventral to GPi) before heading toward the LHb. Stimulation at the areas ventromedial to the GPe-GPi border would activate efferent axons of GPb neurons and induce strong effects on LHb neurons.

### VP-LHb connection

The VP was another structure from which short latency effects were evoked in LHb neurons (Figure 2B). Anatomically, the VP is known to project to the LHb among several other areas (Haber and Knutson, 2010). Berridge and colleagues have suggested that the VP may serve as a ‘limbic final common pathway’ for processing of reward (Smith et al., 2009), a conclusion based on studies using various methods: lesions (Cromwell and Berridge, 1993), inactivations (Farrar et al., 2008), chemical manipulations (Stratford et al., 1999; Shimura et al., 2006), neuronal recordings (Wheeler and Carelli, 2006; Tindell et al., 2006). Human imaging studies are largely consistent with this conclusion (Simmons et al., 2013). A recent study from our lab (Tachibana and Hikosaka, 2012) showed that primate VP neurons encoded expected reward values and costs by changing the level of tonic activity in a stepwise manner. Importantly, a majority of VP neurons were excited by the expectation of a large reward (i.e., positive value coding). Since some VP neurons projecting to the mediodorsal thalamus (Zahm et al., 1987) and the substantia nigra (Bevan et al., 1996) are GABAergic and inhibitory, the VP-LHb connection may also be inhibitory. If so, their positive value coding would be converted to negative value coding in LHb neurons, which is consistent with how LHb neurons behave (Matsumoto and Hikosaka, 2007). However, there is a crucial difference in information processing between VP neurons and LHb neurons: VP neurons represent the expected reward value per se, whereas LHb neurons represent a difference between the actual and expected rewards (i.e., reward prediction error). How the conversion of the information occurs via the VP-LHb connection remains to be solved.

### Striatum-LHb connections

We found that stimulations in the striatum induced diverse responses in LHb neurons. The effect of striatal stimulation was excitatory or inhibitory, depending on where the stimulation was applied in the striatum (Figures 2, 3). Inhibitory effects were evoked from the ventral part of the putamen (Figure 3B) and the ventral striatum (Figure 3A). Excitatory effects were evoked from the dorsal part of the putamen (Figures 3A, B) and the CN (Figure 3C). How can we explain these different effects?

**Figure 4:**
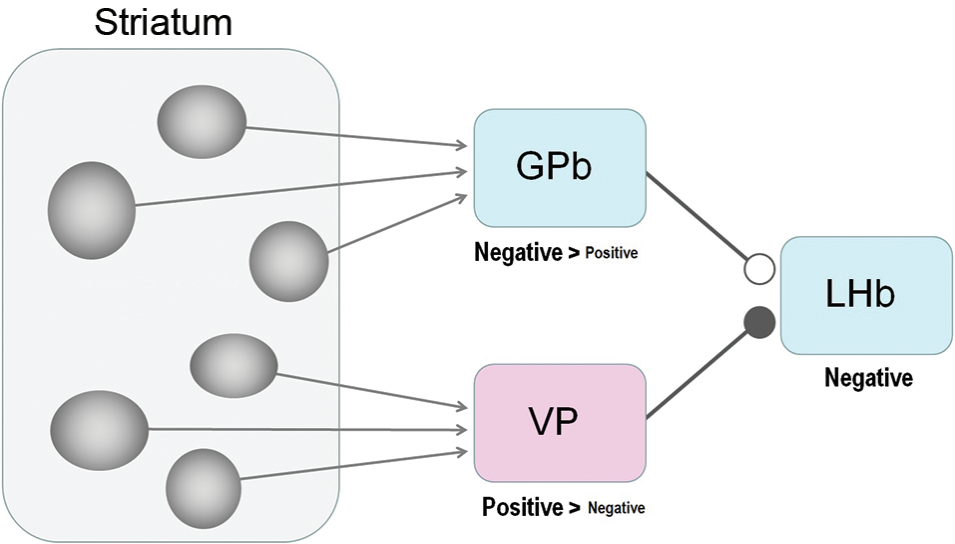
**Striatum-LHb connections: possible circuits through GPb or VP.** Our results suggest that the GPb-LHb connection is excitatory while the VP-LHb connection is inhibitory. A majority of GPb and LHb neurons encode negative reward prediction errors (e.g., excited by the unpredicted omission of reward), whereas a majority of VP neurons encode positive reward values (e.g., excited by the prediction of reward), The excitatory-inhibitory nature is unknown for the striatum-GPb and the striatum-VP connections; the sources of these connections might be the striosomes.

The latencies of these effects are longer than those from the GPb or VP, consistent with anatomical findings indicating the striatum-LHb connection is indirect (Saleem et al., 2002). It is conceivable that the effects were mediated by the GPb (Rajakumar et al., 1993) or the VP (Haber et al., 1993; Figure 4). Whether the net effect is excitatory or inhibitory would thus depend on the nature of the disynaptic (or multi-synaptic) connections.

Medium spiny neurons comprise the only class of output neurons in the striatum and are GABAergic inhibitory neurons (Tepper and Bolam, 2004). It is then expected that striatal stimulations induce inhibitory responses in GPb and VP neurons. Indeed, Tremblay and Filion (1989) found in the monkey that the ordinary types of GPe and GPi neurons were first inhibited by striatal stimulations. On the other hand, what they called ‘border neurons’ were often (but not always) excited by striatal stimulations, possibly because the signals were mediated through inhibitory axon collaterals within the striatum (Tremblay and Filion, 1989) or GPe (Sadek et al., 2007). The border neurons were distributed in the border regions that surround the GPi as well as the GPe, and showed tonic firing whose frequency was lower than the common types of GPi or GPe neurons (DeLong, 1971). Some of the border neurons may correspond to GPb neurons projecting the LHb (Parent et al., 2001). Assuming that the GPb-LHb connection is excitatory (Hong and Hikosaka, 2008a; Shabel et al., 2012; Stephenson-Jones et al., 2013), the net effect of the striatal stimulation would be excitatory (if GPb neurons are excited) or inhibitory (if GPb neurons are inhibited). Therefore, both the excitatory and inhibitory effects caused by striatal stimulations (Figures 2, 3) could be mediated by GPb neurons.

Compared with the striatum-GPb-LHb circuit, the significance of the striatum-VP-LHb circuit is less clear. Anatomically, the VP is known to receive inputs mainly from the ventral portion of the striatum (Heimer et al., 1987; Usuda et al., 1998) and projects to the LHb (Haber et al., 1993). Although it is commonly thought that the striatum-VP connection is GABAergic and inhibitory (Walaas and Fonnum, 1979), it might not apply to VP neurons that project to the LHb. Therefore, it is unclear which part of the striatum modulates the activity of LHb neurons through the VP or through the GPb.

A remarkable feature of the effects of the striatum stimulation, regardless of the underlying synaptic mechanisms, is that the effective sites are distributed in a patchy manner. It has long been known that the striatum is composed of two chemically defined regions, forming patchy structures (called “striosome”) and surrounding areas (called “matrix”) (Graybiel and Ragsdale, 1978; Gerfen, 1984). We speculate that the patchy effective sites in the striatum (Figure 3) may correspond to striosomes, as suggested by Rajakumar et al. (1993). Striosomes receive inputs from limbic areas, such as the amygdala (Gerfen, 1984; Ragsdale and Graybiel, 1988) and the orbitofrontal-insular cortices (Eblen and Graybiel, 1995), suggesting that they carry emotional or motivational signals. Consistent with this idea, rats quickly learn to self-stimulate the striosomal regions, but not matrix regions (White and Hiroi, 1998). Considering this reinforcing effect of the striosome, and the patchy distribution of the striatal stimulation effects on LHb neurons described above, we speculate that the strisome-GPb-LHb circuit contributes to the formation of negative reward prediction error signals in the LHb.

## Conflict of interest statement

The authors declare that the research was conducted in the absence of any commercial or financial relationships that could be construed as a potential conflict of interest.

## References

[B1] Ambroggi F. Ghazizadeh A. Nicola S. M. Fields H. L. (2011). Roles of nucleus accumbens core and shell in incentive-cue responding and behavioral inhibition. J. Neurosci. 31, 6820–6830 10.1523/jneurosci.6491-10.2011 21543612 PMC3145462

[B2] Barroso-Chinea P. Rico A. J. Perez-Manso M. Roda E. Lopez I. P. Luis-Ravelo D. (2008). Glutamatergic pallidothalamic projections and their implications in the pathophysiology of Parkinson’s disease. Neurobiol. Dis. 31, 422–432 10.1016/j.nbd.2008.05.019 18598767

[B3] Barrot M. Sesack S. R. Georges F. Pistis M. Hong S. Jhou T. C. (2012). Braking dopamine systems: a new GABA master structure for mesolimbic and nigrostriatal functions. J. Neurosci. 32, 14094–14101 10.1523/jneurosci.3370-12.2012 23055478 PMC3513755

[B58] Bevan M. D. Smith A. D. Bolam J. P. (1996). The substantia nigra as a site of synaptic integration of functionally diverse information arising from the ventral pallidum and the globus pallidus in the rat. Neuroscience 75, 5–12 10.1016/0306-4522(96)00377-6 8923517

[B4] Brown R. M. Short J. L. Lawrence A. J. (2010). Identification of brain nuclei implicated in cocaine-primed reinstatement of conditioned place preference: a behaviour dissociable from sensitization. PLoS One 5:e15889 10.1371/journal.pone.0015889 21209913 PMC3012115

[B5] Christoph G. R. Leonzio R. J. Wilcox K. S. (1986). Stimulation of the lateral habenula inhibits dopamine-containing neurons in the substantia nigra and ventral tegmental area of the rat. J. Neurosci. 6, 613–619 3958786 10.1523/JNEUROSCI.06-03-00613.1986 PMC6568453

[B6] Cromwell H. C. Berridge K. C. (1993). Where does damage lead to enhanced food aversion: the ventral pallidum/substantia innominata or lateral hypothalamus? Brain Res. 624, 1–10 10.1016/0006-8993(93)90053-p 8252379

[B7] DeLong M. R. (1971). Activity of pallidal neurons during movement. J. Neurophysiol. 34, 414–427 4997823 10.1152/jn.1971.34.3.414

[B8] Eblen F. Graybiel A. M. (1995). Highly restricted origin of prefrontal cortical inputs to striosomes in the macaque monkey. J. Neurosci. 15, 5999–6013 7666184 10.1523/JNEUROSCI.15-09-05999.1995 PMC6577677

[B9] Farrar A. M. Font L. Pereira M. Mingote S. Bunce J. G. Chrobak J. J. (2008). Forebrain circuitry involved in effort-related choice: injections of the GABAA agonist muscimol into ventral pallidum alter response allocation in food-seeking behavior. Neuroscience 152, 321–330 10.1016/j.neuroscience.2007.12.034 18272291 PMC2668809

[B10] Gerfen C. R. (1984). The neostriatal mosaic: compartmentalization of corticostriatal input and striatonigral output systems. Nature 311, 461–464 10.1038/311461a0 6207434

[B11] Graybiel A. M. Ragsdale C. W. Jr. (1978). Histochemically distinct compartments in the striatum of human, monkeys, and cat demonstrated by acetylthiocholinesterase staining. Proc. Natl. Acad. Sci. U S A 75, 5723–5726 10.1073/pnas.75.11.5723 103101 PMC393041

[B12] Haber S. N. Knutson B. (2010). The reward circuit: linking primate anatomy and human imaging. Neuropsychopharmacology 35, 4–26 10.1038/npp.2009.129 19812543 PMC3055449

[B13] Haber S. N. Lynd-Balta E. Mitchell S. J. (1993). The organization of the descending ventral pallidal projections in the monkey. J. Comp. Neurol. 329, 111–128 10.1002/cne.903290108 8454722

[B14] Heimer L. Zaborszky L. Zahm D. S. Alheid G. F. (1987). The ventral striatopallidothalamic projection: I. The striatopallidal link originating in the striatal parts of the olfactory tubercle. J. Comp. Neurol. 255, 571–591 10.1002/cne.902550409 3029188

[B15] Hikosaka O. (2010). The habenula: from stress evasion to value-based decision-making. Nat. Rev. Neurosci. 11, 503–513 10.1038/nrn2866 20559337 PMC3447364

[B16] Hikosaka O. Sakamoto M. Miyashita N. (1993). Effects of caudate nucleus stimulation on substantia nigra cell activity in monkey. Exp. Brain Res. 95, 457–472 10.1007/bf00227139 8224072

[B17] Hikosaka O. Sakamoto M. Usui S. (1989). Functional properties of monkey caudate neurons. III. Activities related to expectation of target and reward. J. Neurophysiol. 61, 814–832 2723722 10.1152/jn.1989.61.4.814

[B18] Hong S. Hikosaka O. (2008a). The globus pallidus sends reward-related signals to the lateral habenula. Neuron 60, 720–729 10.1016/j.neuron.2008.09.035 19038227 PMC2638585

[B19] Hong S. Hikosaka O. (2008b). Convergent inputs from the ventral striatum and the dorsal striatum to the lateral habenula in the monkey. Soc. Neurosci. Abstr. 34:5786.

[B20] Hong S. Jhou T. C. Smith M. Saleem K. S. Hikosaka O. (2011). Negative reward signals from the lateral habenula to dopamine neurons are mediated by rostromedial tegmental nucleus in primates. J. Neurosci. 31, 11457–11471 10.1523/jneurosci.1384-11.2011 21832176 PMC3315151

[B21] Hori Y. Minamimoto T. Kimura M. (2009). Neuronal encoding of reward value and direction of actions in the primate putamen. J. Neurophysiol. 102, 3530–3543 10.1152/jn.00104.2009 19812294

[B22] James M. H. Charnley J. L. Flynn J. R. Smith D. W. Dayas C. V. (2011). Propensity to ‘relapse’ following exposure to cocaine cues is associated with the recruitment of specific thalamic and epithalamic nuclei. Neuroscience 199, 235–242 10.1016/j.neuroscience.2011.09.047 21985936

[B23] Jhou T. C. Geisler S. Marinelli M. Degarmo B. A. Zahm D. S. (2009). The mesopontine rostromedial tegmental nucleus: a structure targeted by the lateral habenula that projects to the ventral tegmental area of Tsai and substantia nigra compacta. J. Comp. Neurol. 513, 566–596 10.1002/cne.21891 19235216 PMC3116663

[B24] Ji H. Shepard P. D. (2007). Lateral habenula stimulation inhibits rat midbrain dopamine neurons through a GABA(A) receptor-mediated mechanism. J. Neurosci. 27, 6923–6930 10.1523/jneurosci.0958-07.2007 17596440 PMC6672239

[B25] Kawagoe R. Takikawa Y. Hikosaka O. (1998). Expectation of reward modulates cognitive signals in the basal ganglia. Nat. Neurosci. 1, 411–416 10196532 10.1038/1625

[B26] Lau B. Glimcher P. W. (2008). Value representations in the primate striatum during matching behavior. Neuron 58, 451–463 10.1016/j.neuron.2008.02.021 18466754 PMC2427158

[B27] Lauwereyns J. Watanabe K. Coe B. Hikosaka O. (2002). A neural correlate of response bias in monkey caudate nucleus. Nature 418, 413–417 10.1038/nature00892 12140557

[B28] Li B. Piriz J. Mirrione M. Chung C. Proulx C. D. Schulz D. (2011). Synaptic potentiation onto habenula neurons in the learned helplessness model of depression. Nature 470, 535–539 10.1038/nature09742 21350486 PMC3285101

[B29] Lisoprawski A. Herve D. Blanc G. Glowinski J. Tassin J. P. (1980). Selective activation of the mesocortico-frontal dopaminergic neurons induced by lesion of the habenula in the rat. Brain Res. 183, 229–234 10.1016/0006-8993(80)90135-3 7357405

[B30] Matsumoto M. Hikosaka O. (2007). Lateral habenula as a source of negative reward signals in dopamine neurons. Nature 447, 1111–1115 10.1038/nature05860 17522629

[B32] Oyama K. Hernádi I. Iijima T. Tsutsui K. (2010). Reward prediction error coding in dorsal striatal neurons. J. Neurosci. 30, 11447–11457 10.1523/jneurosci.1719-10.2010 20739566 PMC6633341

[B33] Parent A. Gravel S. Boucher R. (1981). The origin of forebrain afferents to the habenula in rat, cat and monkey. Brain Res. Bull. 6, 23–38 10.1016/s0361-9230(81)80066-4 7470948

[B34] Parent M. Lévesque M. Parent A. (2001). Two types of projection neurons in the internal pallidum of primates: single-axon tracing and three-dimensional reconstruction. J. Comp. Neurol. 439, 162–175 10.1002/cne.1340 11596046

[B35] Ragsdale C. W. Jr. Graybiel A. M. (1988). Fibers from the basolateral nucleus of the amygdala selectively innervate striosomes in the caudate nucleus of the cat. J. Comp. Neurol. 269, 506–522 10.1002/cne.902690404 2453535

[B36] Rajakumar N. Elisevich K. Flumerfelt B. A. (1993). Compartmental origin of the striato-entopeduncular projection in the rat. J. Comp. Neurol. 331, 286–296 10.1002/cne.903310210 8509503

[B37] Sadek A. R. Magill P. J. Bolam J. P. (2007). A single-cell analysis of intrinsic connectivity in the rat globus pallidus. J. Neurosci. 27, 6352–6362 10.1523/jneurosci.0953-07.2007 17567796 PMC6672453

[B38] Saleem K. S. Pauls J. M. Augath M. Trinath T. Prause B. A. Hashikawa T. (2002). Magnetic resonance imaging of neuronal connections in the macaque monkey. Neuron 34, 685–700 10.1016/s0896-6273(02)00718-3 12062017

[B39] Sartorius A. Kiening K. L. Kirsch P. von Gall C. C. Haberkorn U. Unterberg A. W. (2010). Remission of major depression under deep brain stimulation of the lateral habenula in a therapy-refractory patient. Biol. Psychiatry 67, e9–e11 10.1016/j.biopsych.2009.08.027 19846068

[B40] Shabel S. J. Proulx C. D. Trias A. Murphy R. T. Malinow R. (2012). Input to the lateral habenula from the basal ganglia is excitatory, aversive, and suppressed by serotonin. Neuron 74, 475–481 10.1016/j.neuron.2012.02.037 22578499 PMC3471532

[B41] Shimura T. Imaoka H. Yamamoto T. (2006). Neurochemical modulation of ingestive behavior in the ventral pallidum. Eur. J. Neurosci. 23, 1596–1604 10.1111/j.1460-9568.2006.04689.x 16553623

[B42] Simmons W. K. Rapuano K. M. Ingeholm J. E. Avery J. Kallman S. Hall K. D. (2013). The ventral pallidum and orbitofrontal cortex support food pleasantness inferences. Brain Struct. Funct. [Epub ahead of print] 10.1007/s00429-013-0511-0 23397317 PMC3676475

[B43] Smith K. S. Tindell A. J. Aldridge J. W. Berridge K. C. (2009). Ventral pallidum roles in reward and motivation. Behav. Brain Res. 196, 155–167 10.1016/j.bbr.2008.09.038 18955088 PMC2606924

[B45] Stamatakis A. M. Stuber G. D. (2012). Activation of lateral habenula inputs to the ventral midbrain promotes behavioral avoidance. Nat. Neurosci. 15, 1105–1107 10.1038/nn.3145 22729176 PMC3411914

[B59] Stephenson-Jones M. Kardamakis A. A. Robertson, B. Grillner, S. (2013). Independent circuits in the basal ganglia for the evaluation and selection of actions. Proc. Natl. Acad. Sci. U S A 110, E3670–E3679 10.1073/pnas.1314815110 24003130 PMC3780871

[B46] Stratford T. R. Kelley A. E. Simansky K. J. (1999). Blockade of GABAA receptors in the medial ventral pallidum elicits feeding in satiated rats. Brain Res. 825, 199–203 10.1016/s0006-8993(99)01239-1 10216189

[B47] Tachibana Y. Hikosaka O. (2012). The primate ventral pallidum encodes expected reward value and regulates motor action. Neuron 76, 826–837 10.1016/j.neuron.2012.09.030 23177966 PMC3519929

[B48] Tepper J. M. Bolam J. P. (2004). Functional diversity and specificity of neostriatal interneurons. Curr. Opin. Neurobiol. 14, 685–692 10.1016/j.conb.2004.10.003 15582369

[B49] Tindell A. J. Smith K. S. Pecina S. Berridge K. C. Aldridge J. W. (2006). Ventral pallidum firing codes hedonic reward: when a bad taste turns good. J. Neurophysiol. 96, 2399–2409 10.1152/jn.00576.2006 16885520

[B50] Tremblay L. Filion M. (1989). Responses of pallidal neurons to striatal stimulation in intact waking monkeys. Brain Res. 498, 1–16 10.1016/0006-8993(89)90394-6 2790460

[B51] Tremblay L. Hollerman J. R. Schultz W. (1998). Modifications of reward expectation-related neuronal activity during learning in primate striatum. J. Neurophysiol. 80, 964–977 9705482 10.1152/jn.1998.80.2.964

[B52] Usuda I. Tanaka K. Chiba T. (1998). Efferent projections of the nucleus accumbens in the rat with special reference to subdivision of the nucleus: biotinylated dextran amine study. Brain Res. 797, 73–93 10.1016/s0006-8993(98)00359-x 9630528

[B53] Walaas I. Fonnum F. (1979). The distribution and origin of glutamate decarboxylase and choline acetyltransferase in ventral pallidum and other basal forebrain regions. Brain Res. 177, 325–336 10.1016/0006-8993(79)90783-2 497834

[B54] Wheeler R. A. Carelli R. M. (2006). The neuroscience of pleasure. Focus on “Ventral pallidum firing codes hedonic reward: when a bad taste turns good”. J. Neurophysiol. 96, 2175–2176 10.1152/jn.00727.2006 16885518

[B55] White N. M. Hiroi N. (1998). Preferential localization of self-stimulation sites in striosomes/patches in the rat striatum. Proc. Natl. Acad. Sci. U S A 95, 6486–6491 10.1073/pnas.95.11.6486 9600993 PMC27819

[B57] Zahm D. S. Zaborszky L. Alheid G. F. Heimer L. (1987). The ventral striatopallidothalamic projection: II. The ventral pallidothalamic link. J. Comp. Neurol. 255, 592–605 10.1002/cne.902550410 3029189

